# Surgical ciliated cyst after a mandibular surgery: a particular case report and review of the literature

**DOI:** 10.1186/s12903-021-01991-5

**Published:** 2021-12-09

**Authors:** Irene Lafuente-Ibáñez de Mendoza, Marta Fernández-Reyes, Antonio Fernández-Arenas, José Manuel Aguirre-Urizar

**Affiliations:** 1 Department of Stomatology II, Faculty of Medicine and Nursery, University of the Basque Country, Barrio Sarriena s/n, 48940 Leioa, Spain; 2 Arenas Clinic, Private Service, Corredera de San Marcos, 37, 23700 Linares, Spain

**Keywords:** Surgical ciliated cyst, Mandible, Growth-factors, Pathogenesis, Review

## Abstract

**Background:**

Surgical ciliated cyst is a rare clinicopathological lesion that appears in patients who undergo maxillofacial surgery. In this report we present a particular mandibular case and we discuss the etiopathogenesis and clinicopathological features of this pathology after reviewing the current literature, as well as the origin of its respiratory epithelial profile.

**Case presentation:**

The patient is a 67-year-old male with an irregular radiolucency in a previously tooth extracted area of the mandible. The histopathological study revealed a cystic lesion with a connective wall with chronic inflammation, partially lined by a ciliated pseudostratified epithelium. PAS and CK19 stains showed the respiratory characteristics of this epithelium and confirmed the final diagnosis of mandibular surgical ciliated cyst.

**Conclusions:**

Surgical ciliated cyst is an uncommon entity associated with maxillofacial surgical procedures with bone and nasal cartilage grafts. In our case, treatment with growth factors present in platelet-rich plasma could explain the respiratory changes observed in the cystic epithelial lining.”

## Background

The surgical ciliated cyst is an uncommon and benign entity described by Kubo in 1927 that occurs in the maxillary bones that have previously been surgically treated (orthognathic surgery, plastic surgery, etc.) [1–3]. This pathology is slightly more frequent in men and usually appears as an asymptomatic radiolucency, with or without swelling [4]. The treatment of the surgical ciliated cyst is simple enucleation, and its recurrence rate is low (20%) [5, 6].

It is believed that the origin of the maxillary cases could derive from rests of sinus mucosa that remain trapped in the bone and maintain their proliferative potential [7]. On the contrary, mandibular cases have been more difficult and controversial to explain since its initial description in 1994 [8].

In this report, we present the first case of a surgical ciliated cyst of the mandible unrelated to a major maxillofacial surgery and we discuss its main etiopathogenic and clinicopathological aspects, as well the etiology of its respiratory epithelial profile.

## Case presentation

A 67-year-old man under treatment with antihypertensives and anticoagulants due to cardiac arrhythmia was referred to a dental private practice complaining of a mandibular pain in the fourth quadrant. He had experienced multiple abscesses associated with a fixed dental prosthesis since 2018.

**Fig. 1:**
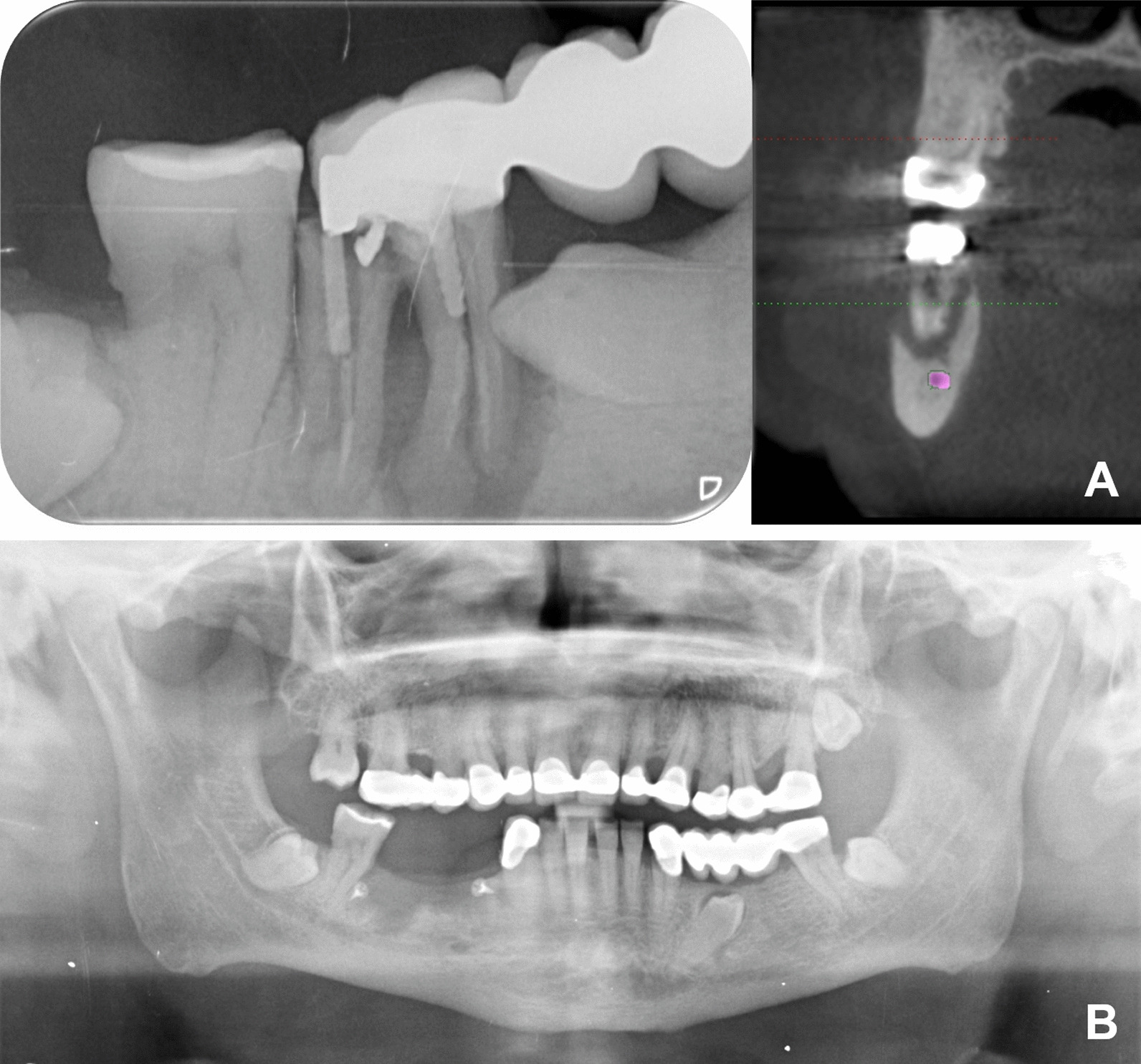
**A** Initial radiographical aspect: Radiolucent endo-periodontal lesion showing fracture of 4.6 with hypercementosis, inclusion of 4.5, and alveolar bone loss. **B** Presurgical radiographical aspect: Irregular radiolucent lesion in the previously operated area

**Fig. 2:**
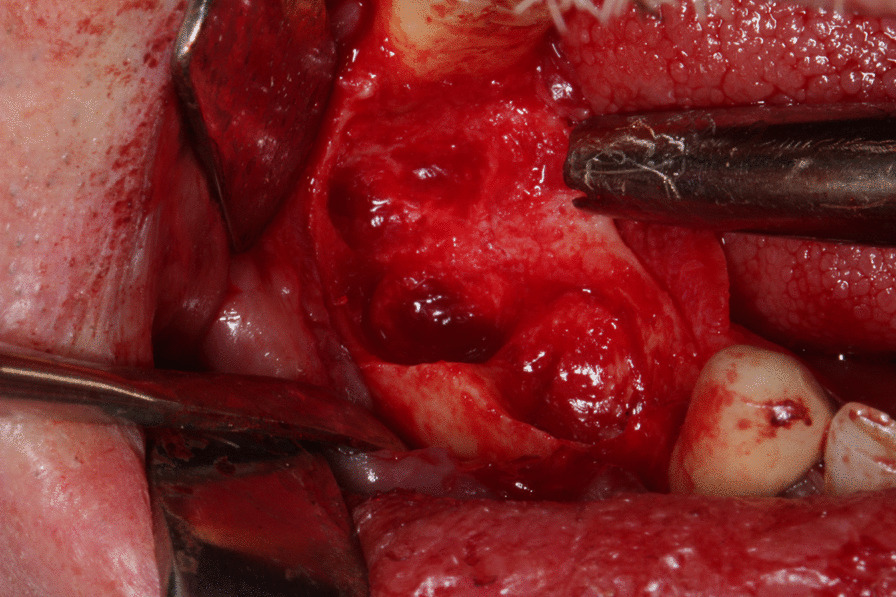
Surgical aspect. Absence of alveolar osseous regeneration and presence of soft cystic lesion

Radiographic examination showed the inclusion of 4.5 and a complex endo-periodontal radiolucent lesion on 4.6, with double root fracture and hypercementosis. CT scan revealed an extensive circumferential alveolar bone loss (Fig. 1A). Under local anesthesia, tooth 4.5 and 4.6 were removed and the alveolar lesion was curetted. The bone defect was filled with platelet-rich plasma and a collagen membrane (Creos Xenoprotec®), fixed with titanium micro-screws (Bioner®). On a follow-up examination, an irregular radiolucency was observed in the surgically treated area two years before (Fig. 1B). Under local anesthesia, a cystic lesion was excised and submitted for histopathologic analysis with the presumptive diagnosis of residual cyst (Fig. 2).

**Fig. 3:**
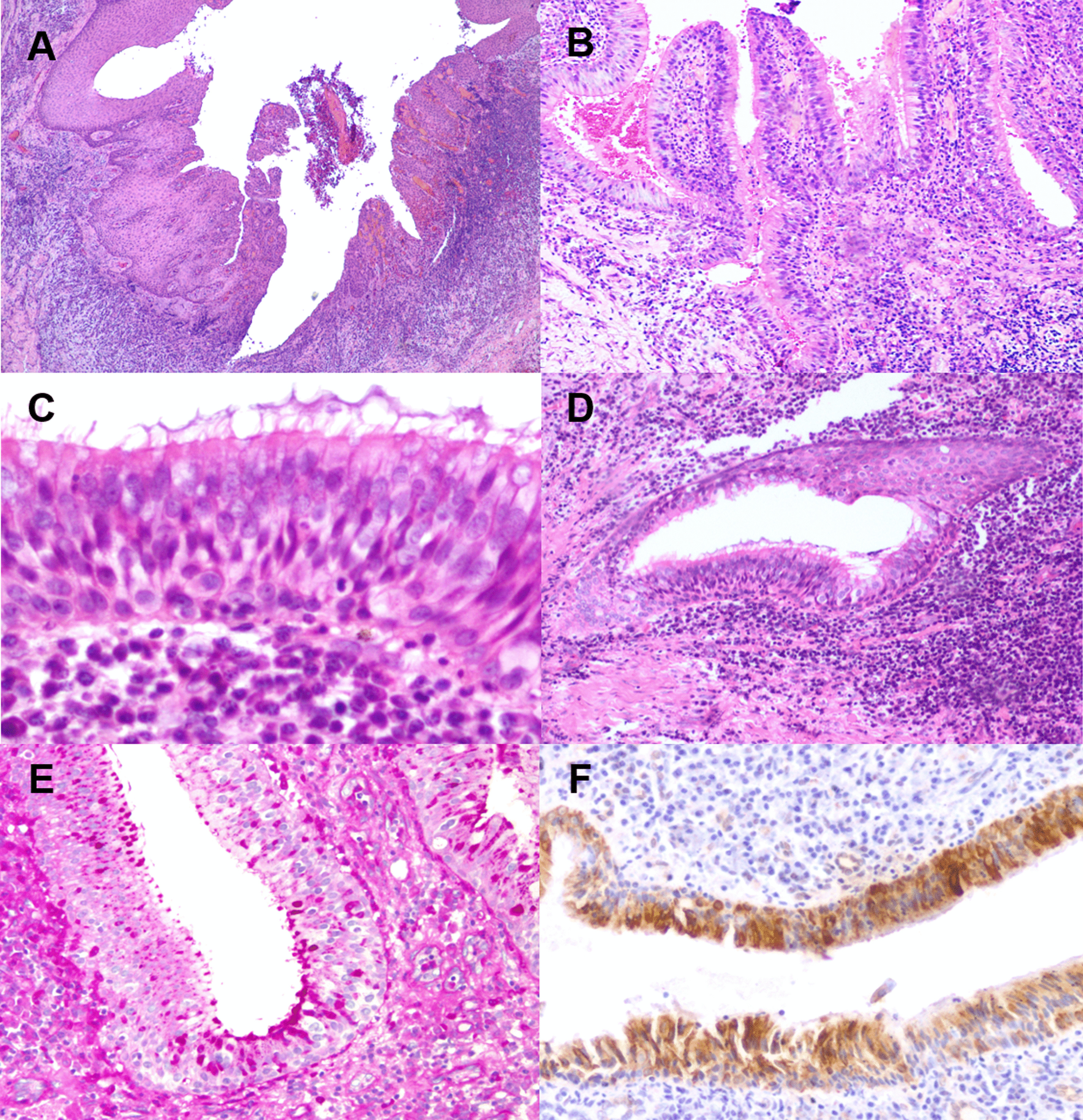
Histopathological features. **A** Connective tissue wall with chronic inflammation, lined by a hyperplastic non-keratinized stratified epithelium with inflammatory exocytosis (10x, H&E); **B** pseudostratified epithelial lining with papillary arrangement (20x, H&E); **C** detail of the pseudostratified ciliated epithelium (40x, H&E); **D** parietal nest with foci of epithelial transition (25x, H&E); E) PAS (+) cells in the metaplastic epithelium (30x); **F** positive immunohistochemical expression of CK19 in the epithelial lining (30x)

The tissue specimen consisted of a dark brown and irregularly-shaped fragment of soft tissue, measuring 1.2 × 1 × 1 cm and brownish cut surface. Microscopic examination showed a thick fibrocollagenous connective tissue wall with different densities and a chronic inflammatory infiltrate (Fig. 3A). The epithelial lining consisted of a non-keratinised and hyperplastic stratified epithelium with inflammatory exocytosis, that presented large areas of ciliated pseudostratified epithelium with papillary foci (Fig. 3B, C). Epithelial nests were observed in the parietal connective tissue, showing the transition between non-keratinised stratified epithelium and ciliated pseudostratified epithelium (Fig. 3D). Positivity for CK19 (RCK108, ThermoFisher, Thermo Fisher Scientific, Waltham, MA ®) and PAS (+) mucous cells confirmed the respiratory profile of the epithelium (Fig. 3E, F).

Based on these data, the final diagnosis of the lesion was surgical ciliated cyst of the mandible. At a one-year of follow-up there was no evidence of recurrence or complications.

## Discussion and conclusions

The prevalence of the surgical ciliated cyst is variable (3–20% of patients with history of maxillofacial surgery), and it is more frequently located in the upper maxilla [9, 10]. The first mandibular case was reported in a woman who underwent chin augmentation with a subperiosteal bone and nasal cartilage graft [8].

After performing a literature review on the surgical ciliated cyst of the mandible, we found 12 clinical cases [2, 8–15] (Table 1). The age of patients ranged from 33 to 59 years (mean: 36.9 ± 13 years), and males and females were equally affected. The diagnosis of these lesions occurred from 2 to 40 years after the surgery, mainly as a swelling on the chin area, or an asymptomatic radiographic finding. In most cases the surgical history was of rhinoplasty, orthognathic surgery or genioplasty (Table 1). Our patient is the first associated to a bone augmentation surgery. The treatment of choice in all reports was complete excision and curettage, and no recurrences have been reported.

**Table 1:** Main clinicopathological features of the mandibular surgical ciliated cyst

References	Patient (age/sex)	Location (Mandible)	Symptoms	Surgery	Evolution (years)
Nastri and Hookey [8]	33/F	Anterior	Swelling, pain	Rhinoplasty, chin augmentation	15
Anastassov and Lee [20]	53/M	Anterior	Swelling	Rhinoplasty, chin augmentation	39
Kelly et al. [21]	56/F	Anterior	Swelling, pain	Rhinoplasty, genioplasty	40
Imholte and Schwartz [22]	59/M	Anterior	Swelling	Rhinoplasty, chin augmentation	40
Koutlas [11]	34/F	–	Swelling	Orthognathic	21
Bourgeois et al. [2]	24/F	–	Radiolucency	Orthognathic, genioplasty	4
Lazar et al. [19]	24/M	–	Swelling, fistula	Rhinoplasty, mentoplasty	5
Ragsdale et al. [12]	30/M	Anterior	Swelling, fistula	Orthognathic	16
Cai et al. [14]	23/M	–	Swelling	Orthognathic, genioplasty	2
Seifi et al. [9]	31/F	Anterior	Swelling	Orthognathic, genioplasty	2
Syyed et al. [15]	38/M	Anterior	Radiolucency	Orthognathic, genioplasty	18
	25/F	Anterior	Swelling	Orthognathic, genioplasty	10
Current case	50/M	Posterior	Radiolucency	Platelet-rich plasma, collagen membrane	> *2*

The presence of respiratory epithelium in the upper maxillary odontogenic cysts (radicular cyst, dentigerous cyst, odontogenic keratocyst) is uncommon [16, 17]. Regarding the surgical ciliated cyst, the use of bone grafts from the maxilla and/or nasal cartilage could explain the presence of the ciliated pseudostratified epithelium [8, 12, 18, 19]. In the cases without grafting, the transplantation of respiratory epithelial rests during the bimaxillary surgical procedure is the etiopathogenic hypothesis more frequently considered [2, 14–16, 20, 21]. Nevertheless, the existence of this particular epithelium is more difficult to explain in cases without a major maxillofacial surgery, as in our patient.

We believe that the platelet-rich plasma (PRP) used for the bone augmentation and the history of a previous chronic severe inflammatory lesion could be the key pathogenic elements of our atypical case. PRP is a concentrated solution containing multiple secreted proteins, such as platelet-derived growth factor (PDGF), transforming growth factor (TGF)-β, endothelial growth factor (VEGF), epidermal growth factor (EGF), fibroblast growth factor (FGF) and insulin-like growth factor (IGF) [23, 24]. It has been reported that some of these mitogenic factors promote proliferation, differentiation and maturation of odontogenic epithelial cells and trigger the formation of a cystic lesion, as well as areas of respiratory metaplasia [25]. On the other hand, we know that certain inflammatory mediators are actively involved in the development of maxillary cystic lesions (interleukins 1, 13) [26]. These proteins lead to the overexpression of cell growth factors, and participate in the proliferation and ciliated metaplasia of the epithelium [27].

In this special case we think that there are 2 histogenetic possibilities that could explain the appearance of the cyst. The first is that the combination of factors from the PRP and the chronic inflammatory response induced the development of a de novo cyst over the mesenchymal alveolus site after the initial surgery, with extensive areas of respiratory epithelial metaplasia. The second is that remnants of the previous endo-periodontal inflammatory odontogenic lesion remained in the alveolus site after the initial surgery, over which the PRP factors and inflammatory proteins acted, inducing their proliferation, growth and respiratory epithelial metaplasia.

In summary, the surgical ciliated cyst of the mandible is an uncommon clinicopathological entity that occurs in patients with history of a maxillofacial surgery, mostly associated with maxillary bone-cartilage grafts. Our case is the first to develop in a patient treated with platelet-rich plasma. Thus, it is very important to make a good clinical history and follow-up of all patients treated surgically in the jaw bones.

## Data Availability

All data generated or analysed during this study are included in this published article [and its supplementary information files].

## Abbreviations

CK: Cytokeratin
CT: Computed tomography
EGF: Epidermal growth factor
FGF: Fibroblast growth factor
IGF: Insulin-like growth factor
PAS: Periodic acid–Schiff
PDGF: Platelet-derived growth factor
PRP: Platelet-rich plasma
TGF-β: Transforming growth factor beta
VEGF: Endothelial growth factor
